# Natural attenuation model and biodegradation for 1,1,1-trichloroethane contaminant in shallow groundwater

**DOI:** 10.3389/fmicb.2015.00839

**Published:** 2015-08-25

**Authors:** Qiang Lu, Rui-Li Zhu, Jie Yang, Hui Li, Yong-Di Liu, Shu-Guang Lu, Qi-Shi Luo, Kuang-Fei Lin

**Affiliations:** ^1^State Environmental Protection Key Laboratory of Environmental Risk Assessment and Control on Chemical Process, School of Resource and Environmental Engineering, East China University of Science and Technology, Shanghai, China; ^2^Shanghai Academy of Environmental Sciences, Shanghai, China; ^3^Shanghai Engineering Research Center of Contaminated Sites Remediation, Shanghai Institute for Design and Research on Environmental Engineering, Shanghai, China

**Keywords:** 1,1,1-trichloroethane, contaminated groundwater, natural attenuation, bacterial communities, dechlorination

## Abstract

Natural attenuation is an effective and feasible technology for controlling groundwater contamination. This study investigated the potential effectiveness and mechanisms of natural attenuation of 1,1,1-trichloroethane (TCA) contaminants in shallow groundwater in Shanghai by using a column simulation experiment, reactive transport model, and 16S rRNA gene clone library. The results indicated that the majority of the contaminant mass was present at 2–6 m in depth, the contaminated area was approximately 1000 m × 1000 m, and natural attenuation processes were occurring at the site. The effluent breakthrough curves from the column experiments demonstrated that the effectiveness of TCA natural attenuation in the groundwater accorded with the advection-dispersion-reaction equation. The kinetic parameter of adsorption and biotic dehydrochlorination of TCA was 0.068 m^3^/kg and 0.0045 d^–1^. The contamination plume was predicted to diminish and the maximum concentration of TCA decreased to 280 μg/L. The bacterial community during TCA degradation in groundwater belonged to *Trichococcus*, *Geobacteraceae*, *Geobacter*, *Mucilaginibacter*, and *Arthrobacter*.

## Introduction

1,1,1-Trichloroethane (TCA) is widely used as a cleaning solvent for degreasing metal parts (Scheutz et al., 2011). Extensive use and improper storage, handling, and disposal as well as its high chemical stability have contributed to TCA being one of the most frequently encountered subsurface contaminants at industrial facilities and waste disposal sites (Aulenta et al., 2006). Furthermore, TCA can threaten potable water resources and natural ecosystems, and the problem could be remediated through physical-chemical and/or biological technologies (Connon et al., 2005). Before remediation technologies are applied to contaminated groundwater, the transport and transformation characteristic of chlorinated aliphatic hydrocarbons (CAHs) in the groundwater must be investigated (Powell et al., 2014).

Chlorinated aliphatic hydrocarbons are often present in the form of dense non-aqueous phase liquids (DNAPLs) in subsurface environments (Puigserver et al., 2013). Migration of these contaminants in the groundwater is affected by dissolution of the pollutants within the source zone, mass transfer to the groundwater, advection, dispersion, diffusion, sorption (retardation), and biodegradation (Schirmer and Butler, 2004). Although conventional treatment technologies such as air-stripping with activated carbon adsorption and pump-and-treat methods (McNab et al., 2000) effectively reduce the risk of contaminant transport away from the source area, their operating costs are often very high due to large energy demand and high treatment costs. In addition, these remedial actions do not usually reduce the duration of the DNAPL source (Viotti et al., 2013). Therefore, to solve these problems, several technologies for degradation of pollutants in the source area have been proposed, including chemical oxidation or reduction, flushing, thermal treatment, anaerobic bioremediation (Grostern and Edwards, 2006; Grostern et al., 2009), and injection of zerovalent iron (Tosco et al., 2012).

While these chemical transformation processes are suitable for source areas, and for control of contaminated plumes, in-situ biological reduction of CAHs may be a valid approach capable of overcoming most of the limitations associated with pump-and-treat methods (Velimirovic et al., 2013). Biologically enhanced reductive dechlorination (ERD) is generally accomplished by injecting an exogenous electron donor into the groundwater to stimulate indigenous bacteria using CAHs as electron acceptors (Löffler and Edwards, 2006; Scheutz et al., 2008). Under anaerobic conditions, 1,1,1-TCA is reductively dechlorinated to 1,1-dichloroethane (DCA), then to chloroethane (CA), and finally to ethane (Scheutz et al., 2011). However, application pilot tests and remedial simulations for TCA are still evolving and facing a number of challenges. For example, while pure and mixed cultures that degrade TCA have been described (Sun et al., 2002; Grostern and Edwards, 2006), rigorous field-scale bioaugmentation tests for treatment of TCA have not been demonstrated. Thus, given these challenges, natural attenuation may have advantages over active bioremediation methods.

Natural attenuation typically relies on adaptation of microorganisms that can use CAHs for growth and reproduction without human interference (Hamonts et al., 2012). Natural attenuation of contaminants in groundwater systems depends on non-destructive physical processes such as dissolution, advection, dispersion, diffusion, and sorption (retardation) and more importantly, on destructive processes such as biodegradation (Scheutz et al., 2011). Therefore, if natural attenuation is being considered for removal of TCA in groundwater, a sound understanding of ongoing processes as well as careful management and monitoring [monitored natural attenuation (MNA)] are required.

Although a feasibility study may provide evidences for natural attenuation, a complete understanding of transport and transformation processes in the aquifer is necessary. The rate of removal should be estimated and the mechanisms of natural attenuation should be analyzed (Rahmawati et al., 2013). Thus, field studies of natural attenuation should be combined with laboratory studies that simulate the groundwater environment using modeling tools that represent natural attenuation and remedial design processes.

The large diversity of modeling applications and number of packed-column experiments described in the literature demonstrated their critical role in enhancing our understanding of the physical, chemical, and hydrodynamic processes governing the transport and remediation of contaminants in groundwater (Matthew and Chrysikopoulos, 2010). However, for reactive transport, experimental columns are typically packed with a loose granular porous medium, such as sand or glass beads; intact soil from a field site or groundwater of interest is less frequently employed. Hence, based on reliable methods, we employed a one-dimensional soil column to simulate transport and natural attenuation using materials from the groundwater.

The spatial and temporal distributions of pollutants in the groundwater are generally assessed through hydrogeologic modeling, which has evolved into a powerful tool that can simulate groundwater flow and contaminant transport at complex sites(Yang et al., 2011). We used visual MODFLOW software v. 4.1 (Schlumberger Inc., New York, NY, USA), which can analyze field-scale transport and biodegradation processes and evaluate, design, plan, and implement effective remediation strategies for contaminated groundwater (Lautz and Siegel, 2006; Yanxun et al., 2011). Further, the role of bacteria was also demonstrated in the degradation of TCA. An anaerobic TCA-degrading mixed bacterial enrichment, including strains of *Dehalobacter* sp. and *Desulfovibrio* sp., was reported to completely degrade TCA (Grostern and Edwards, 2006; Grostern et al., 2009).

The purpose of this study was to demonstrate an integrated method for characterizing migration and natural attenuation of 1,1,1-TCA at contaminated sites. Field investigations provided a preliminary indication that natural attenuation could be a viable remedial strategy. The soil column experiment simulated contaminant transport and fate in groundwater to illustrate processes affecting the contaminant plume and to determine absorption and degradation parameters for modeling. Using the Visual MODFLOW software and calculated parameters from the column experiment, a numerical model was established to better understand the future development of the plume and associated impacts for management and monitoring. Finally, the preponderant bacterial community species were confirmed to elucidate TCA biodegradation in groundwater.

## Materials and Methods

### Site Description and Sample Collection

**FIGURE 1:**
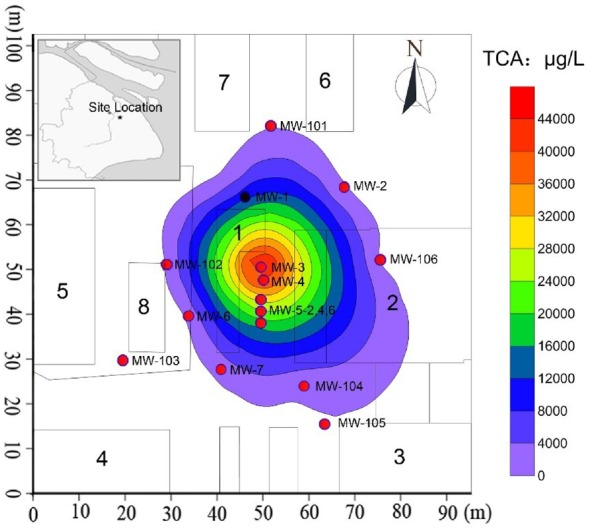
**Details of TCA contamination plume and monitoring network in Area-4 site.** The number 1 is the chemical warehouse; the number 2–6 are workshop; the number 7–8 are product warehouses; MW-1 to MW-7 and MW-101 to MW-106 are the monitoring wells.

The TCA contaminated site was located in a factory, Pudong New District, Shanghai, China. The factory had used chlorinated solvents to degrease petroleum hydrocarbons from the surface of metal products. In this study, the contaminated area investigated (the Area-4 site) was a chemical warehouse in the western part of the industrial site, where new and spent TCA drums were stored directly on the asphalt outside of the chemical warehouse without cover and/or secondary containment. Figure 1 showed the surface features of the Area-4 site and the locations of the monitoring wells placed throughout the area to monitor contaminant concentrations and groundwater levels. Groundwater samples were collected from the monitoring wells for analysis and modeling. In addition, soil from a depth of 3–6 m below ground surface (bgs), was identified as gray sandy silt. All materials were transported to the lab and stored at 4°C under a 100% nitrogen atmosphere until the analysis.

### Chemical Analysis

After collection, 300 μL of the groundwater samples were mixed with 900 μL n-hexane. After oscillation and extraction, 1 μL of the organic phase was analyzed by a gas chromatograph (GC7890A; Agilent Technologies, Santa Clara, CA, USA) equipped with a flame ionization detector and a DB-VRX capillary column (60 m × 0.25 mm × 1.4 μm). N_2_ (ultrapure) served as the carrier at flow rate of 5 mL/min, respectively. The flow rates of H_2_ and air were 40 mL/min and 300 mL/min, respectively. The temperatures of injector, oven, and detector were 240°C, 75°C, and 260°C, respectively. Samples were injected using a split ratio of 20:1. In addition, the NaCl conductivity of the samples in the non-reactive tracer experiments was measured using a conductivity meter (DDS-307A; Leici, Shanghai, China). The pH of the solution was measured using a pH meter (PHS-3C; Leici).

### Groundwater Field Analysis

The velocity field of the groundwater in the shallow groundwater was simulated using MODFLOW with a full 3D approach (Wang et al., 2008). The total extent of the Area-4 site was 100 m × 100 m and the adopted discretization was based on a quadratic mesh (Dx = –Dy = 1.0 m, four layers). The boundary conditions for flow were constant head boundaries determined from values measured for the water table in the observation wells in June 2007. To verify the groundwater model, calibration of modeled groundwater flow conditions was performed using the water levels measured for the shallow groundwater during March 2008 as calibration targets. The groundwater model was decoupled from the reactive transport model.

### Column Simulation Analysis

The experimental columns(Chen et al., 2013), which were sterilized before usage, were designed to simulate the contaminated groundwater and assess the effectiveness of natural attenuation of TCA. The influent solution was continuously pumped from the influent reservoir, a modified 6 L borosilicate glass vessel with an N_2_ pocket, to the bottom of the column via a peristaltic pump. The column consisted of a 10 cm (inside diameter) × 30 cm (length) borosilicate glass columns with an effluent sampling port at the top.

In the column experiment, Column #1 was designed to simulate the contaminated groundwater at the site by using a TCA influent solution with groundwater from the Area-4 site (Table 1), Column #2 served as a control and its influent solution was TCA in sterilized deionized water. The packing material (aquifer soil) was dried and pulverized before packing. The columns were packed with sterile packing material prepared under anoxic, water-saturated conditions and operated in an upflow mode at 20–25°C. Detailed parameters for the two finished columns were shown in Table 2.

**TABLE 1:** **Chemical parameters of influent solution**.

	**Cl^–^ (mg/L)**	**NO_3_^–^ (mg/L)**	**SO_4_^2–^ (mg/L)**	**Fe^2+^ (mg/L)**	**Ca^2+^ (mg/L)**	**Mg^2+^ (mg/L)**	**pH**	**TCA (mg/L)**	**DCA (mg/L)**
#1	3976.20	0.00	243.6	0.21	0.24	0.02	7.3	31.36 ± 1.87	11.25 ± 1.34
#2	0.00	0.00	0.00	0.00	0.00	0.00	6.8	31.36 ± 1.87	0.00

**TABLE 2:** **Column experimental parameters**.

**Parameters**	**Value**
Porosity	0.296(#1), 0.289(#2) (Suchomel et al., 2007)
Median grain size *d*_50_, mm	0.06 (Suchomel et al., 2007)
Volume weight, g/cm^3^	1.85(#1), 1.88(#2) (Amos et al., 2009)
total organic carbon *f*_*oc*_, %	0.084^a^
Vertical hydraulic conductivity, m/s	2.25 × 10^–7b^
Intrinsic permeability, m^2^	2.39 × 10^–14b^
Total pore volume, cm^3^	697, 681 (Amos et al., 2009)

^*a*^Measured by organic carbon analyzer (TOC-VCPN, Shimadzu, Japan). ^*b*^Measured by permeability apparatus (TST-70, Ningxi, China).

During the first stage of the experiment, mineral salts in the column media were reduced by simultaneously flushing deionized water through the two columns in an upflow mode at 0.25 mL/min for 2.0 pore volumes (PV) using a peristaltic pump (BT601L; Baoding Leifu, Baoding, China) with viton tubing in the pump head. Following this mineral salt removal phase, 3 g/L NaCl solution as a tracer material was delivered at a flow rate of 0.1 mL/min for 3.0 PV. The breakthrough curves (BTCs) for NaCl in the effluent samples were determined by reference to the curve for measured conductivity versus concentration of the NaCl solution. After the BTC for the NaCl solution was obtained, the columns were flushed with deionized water at 0.25 mL/min for 2.0 PV. The groundwater from the Area-4 site was then pumped into Column #1 at a same rate for 3.0 PV, while deionized water was used in Column #2. Each column was then operated 25 days with an influent solution containing 31.36 ± 1.87 mg/L TCA stored in the influent reservoir, and the flow rate was reduced to 0.1 mL/min to increase the solution residence time within the column and minimize disruption of flow within the column. Effluent samples (20 mL) for chlorinated ethanes and microbial analyses were collected using syringe from the effluent collection cell every 2 days.

### Reactive Transport Model

Reactive transport modeling was carried out in a 3D mode using the MT3D code for analyzing the migration and transformation of TCA (Prommer et al., 2003). The resulting groundwater velocity field for the aquifer was used as an input to the reactive transport model and TCA concentrations at the Area-4 site in June 2007 were chosen as the initial conditions. Related parameters (e.g., hydraulic conductivity and kinetic parameter of adsorption) used in the simulations were obtained from the laboratory column experiments and field investigations. The model was run to predict the transport and transformation of TCA over space and time, and the results were compared with the sampling results from related wells to evaluate the accuracy of the formulated model.

### Cloning, Sequencing, and Phylogenetic Analysis

Two milliliters of sample were collected from groundwater in monitoring well MW-5–4 m and filtered with the 0.22 μm sterile membrane. Total DNA was extracted from each sample by the bead beating technique (BioSpec, Shanghai, China) using the kit UltraClean DNA Isolation (Mo Bio Laboratories, BIOzym, Landgraaf, Netherlands). Polymerase chain reaction (PCR) amplification was implemented with bacterial primers 27F and 1492R (Li et al., 2015), using a 25 μL reaction mixture containing the template 0.5 μL, each primer (10 mmol/L) 0.5 μL, TaqMix 12.5 μL, and ddH_2_O 11 μL. PCR was performed under the cycling conditions described as follows: initial denaturation at 94°C for 5 min, 25 cycles of denaturation at 94°C for 1 min, annealing at 50°C for 30 s, and extension at 72°C for 1 min, then re-extension at 72°C for 10 min. The PCR reactions were performed with a Mastercycler gradient (Eppendorf, New York). The PCR products were purified with a DNA purification kit (Qiagen, Hilden, Germany) and then ligated into the pMD 19-T vector system (Takara, Dalian, China). The combined plasmids were transformed into *E. coli* DH5α. The insertion of the 16S rRNA gene was screened and retrieved by PCR amplification with the primer set M13–47 and RV-M (Li et al., 2013) The positive clones were sequenced (Sangon, Shanghai, China). After editing and checking sequences manually, typical sequences were identified to closest relatives by the BLAST software in the GenBank database of NCBI. Phylogenetic trees were constructed by Clustal X (2.0) and MEGA (5.05). Robustness for individual branches was estimated by bootstrapping based on 1000 replications.

### Nucleotide Sequence Accession Numbers

The sequences determined in this study were deposited in the GenBank databases under accession numbers: KJ149798–KJ149802 for TCA1–TCA5.

## Results and Discussion

### Investigation of Contamination Plume in Groundwater

Groundwater contamination investigations were conducted at the site between September 2007 and April 2013. Groundwater samples collected from the monitoring wells were submitted for analysis of 1,1,1-TCA and 1,1-DCA by GC. Figure 1 presented a summary of analytical results for four groundwater samples for June 2007. Based on these data and the locations of the samples (Figure 1), the location of the source area and the distribution of 1,1,1-TCA in space were estimated. Horizontally, CAHs were not detected in groundwater samples collected from MW-102 (south), MW-101 (west), or MW-106 (north) in June 2007. 1,1,1-TCA was detected in groundwater from MW-104 at a concentration of 297 μg/L, marginally below the Dutch intervention value (DIV) of 300 μg/L. 1,1-DCA was detected in a groundwater sample from MW-4 at a concentration of 11.7 μg/L, well below the DIV of 900 μg/L. The highest concentrations of 1,1,1-TCA were detected in groundwater samples, collected from MW-3 (49.70 mg/L) and MW-5 (22.00 mg/L in MW-5–4 m), both of which were the likely source area (DNAPL) of the large chlorinated solvent plume. Vertically, analysis of groundwater samples collected from MW-3 (screened at 2–4 m bgs), MW-5 (screened at 4–6 m bgs), and MW-4 (screened at 6–8 m bgs) located in the source area, indicated that the majority of the contaminant mass was present at about 2–6 m bgs (silty clay/clayey silt and sandy silt layer). In addition, based on the distances to the wells outside the source area (Figure 1), the area impacted by 1,1,1-TCA was approximately 1000 m × 1000 m.

The velocity field for the shallow groundwater indicated that some higher-than-expected measured groundwater elevations were likely to be affected by perched water due to leakage and did not accurately represent the actual shallow groundwater elevation. However, the model was considered to be sufficiently well calibrated. The model results showed that the velocity field for groundwater relative to contaminant transport was stable and its hydraulic slope was low.

### The Model of Transport and Natural Attenuation of TCA Contaminant

The BTCs, assuming that transformation of TCA in the aquifer conformed to a first-order reaction kinetics model (Toride et al., 1995; Prommer et al., 2003), were achieved in the equation for advective-dispersion-reaction (ADR) with adsorption and degradation in the one-dimensional form. The governing equation elucidated transport of organic pollutants in one dimension. The absorption and degradation parameters for modeling were estimated using the measured BTC data from the solution of the equation:

(1) $$\frac{\partial \theta c}{\partial t}=D\frac{\partial }{\partial x}\left(D\theta \frac{\partial c}{\partial x}\right)-\frac{\partial c}{\partial x}\left(vC\right)-n_{s}\rho_{s}\frac{\partial s}{\partial t}-Cexp\left(-kt\right)$$

**FIGURE 2:**
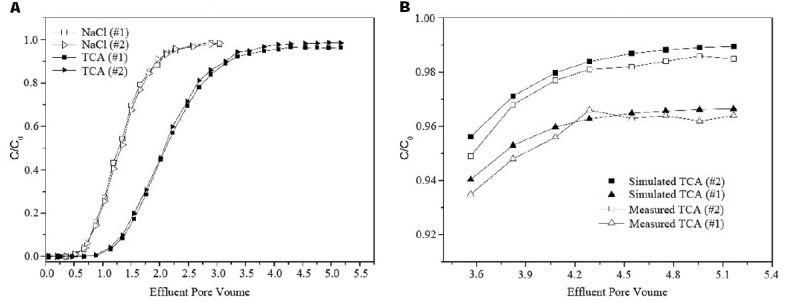
**The effluent breakthrough curves in column experiments. (A)** Effluent concentration of 1,1,1-TCA and NaCl. **(B)** Simulated and Measured curves of 1,1,1-TCA.

Two independent experiments were performed to characterize the hydrodynamic properties of the packed columns and migration and transformation of TCA. The effluent BTCs obtained from both the non-reactive tracer and TCA solution experiments were shown in Figure 2A. The trends and differences in the BTCs indicated the migration and transformation processes of TCA in the column. The BTCs from the NaCl experiments were nearly the same for the two independent columns, as they were similar in terms of environmental properties. However, in the two packed columns with nearly the same hydrodynamic properties, the two BTCs for the TCA solution were significantly different. Compared with Column #2 as the control, the relative concentrations of TCA in Column #1 were lower, and the difference became larger before the effluent concentration was stable. Between 4.75 and 5.16 PV, the average difference in concentration between the columns was 0.68 mg/L. Considering natural attenuation at the TCA-contaminated site, a TCA dechlorination microbial culture was present in the groundwater in Column #1 that accounted for the difference.

When the kinetic parameter of adsorption was 0.068 m^3^/kg, the simulated BTC for TCA (*x* = 40 cm) in Column #1 was similar to the experimental BTC due to the difference resulted from the sampling and dilution. Based on the results of the Column #2 experiment, the kinetic parameter for biotic dehydrochlorination of TCA (*k_TCA_*) was 0.003–0.006 d^–1^. In addition, the simulated BTC for TCA between 3.35 and 5.16 PV when the *k_TCA_* was set to 0.0045 d^–1^ was shown in Figure 2B.

### Prediction of Spatial and Temporal Distributions of TCA Contaminant

**FIGURE 3:**
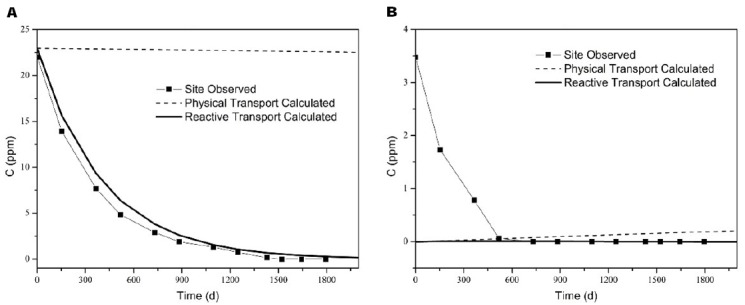
**Comparison between the observed and calculated concentrations [(A) MW-5–4 m (MSE = 1.29), (B) MW-5–6 m (MSE = 1. 96)]**.

A comparison between the observed and calculated concentrations in the selected monitoring wells was made to evaluate the accuracy of the simulation results, and the results were shown in Figure 3. The calculated values of the mean squared errors (MSE) for TCA were also obtained for calibration. The MSE for the high concentration point (MW-5–4 m) was smallest, while for low concentration points, particularly MW-5–6 m, the MSE was large. These significant differences in the values were mainly due to sampling error and the variability of the results for the aquifer in high-concentration areas that were influenced by the source of contaminations. However, the simulated trend for reactive transport among the points was consistent with the trend in groundwater, and this result indicated that calculated transport and natural attenuation reflected the actual situation in the influenced aquifer. Physical transport (no transformation) was to be limited, due to the stability of the water level.

**FIGURE 4:**
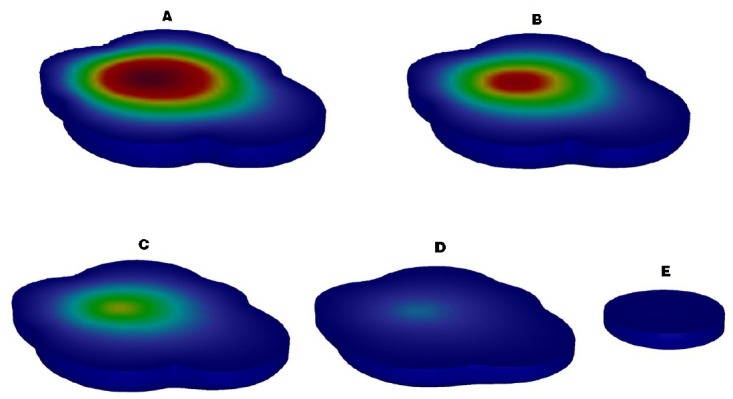
**Description of the contaminant plume of TCA in spatial and temporal [(A) Time 1 d, (B) Time 183 d, (C) Time 365 d, (D) Time 730 d, (E) Time 1825 d]**.

A 3D reactive transport model with no persistent source was successfully developed and calibrated with the *k_TCA_* set to 0.0045 d^–1^. The location of the source area and the distribution of the contaminant plume based on field measurements in June 2007 were replicated in detail (Figure 4). In addition, the ongoing evolution of the plume was modeled for management and remediation project design. Under natural conditions, the modeled contaminant plume was fully representative of the time course of the plume in the aquifer. The source area (Figure 4, red section, C ≥ 25 mg/L) disappeared after about 1 year, due to the characteristics of the contaminated aquifer. After about 5 year, the area contaminated with TCA had diminished and the maximum concentration of TCA was 280 μg/L, below the DIV of 300 μg/L.

### TCA-Degrading Bacterial Community in Groundwater

**FIGURE 5:**
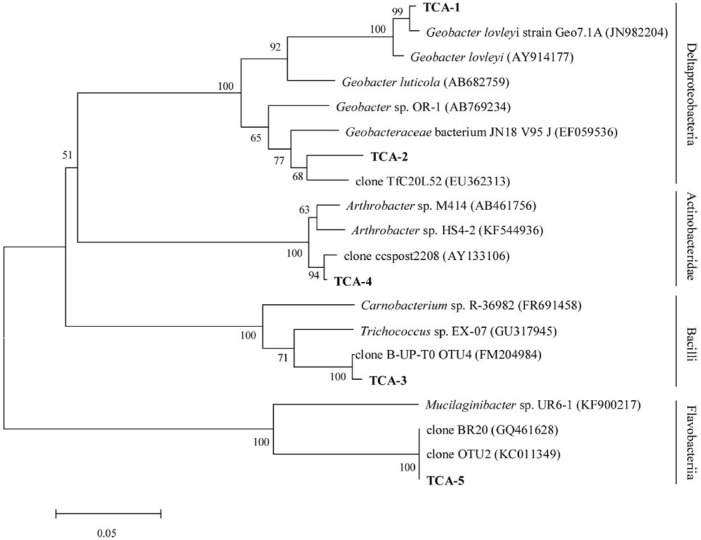
**Phylogenetic tree of the V6 partial sequence of bacterial 16S rRNA from the bacteria in groundwater shown in boldface and the referred sequences in the EMBL database with the putative divisions listed to the right.** The topology show was calculated with the Neighbor-joining method. Bootstrap values *n* = 1000 replicates. Of ≥50% were reported near the corresponding nodes. The scale bar represented 0.05 nuclear acid substitutions per nucleotide position.

The degradation community was the primary reason for reductions in contamination in groundwater and its structure was investigated using a clone library. A total of 34 positive clones were clustered into five operational taxonomic unit (OTUs) at 97% similarity by Mothur 1.32. The rarefaction curve flattened to the right, demonstrating that a reasonable individuals in the community were sampled. The phylogenetic tree showed that the dominant enriched species fell into four major groups: Deltaproteobacteria (13/34, 38.24%, two OTUs), Bacilli (16/34, 47.06%, one OTU), Actinobacteridae (2/34, 5.88%, one OTU), and Flavobacteriia (3/34, 8.82%, one OTU; Figure 5). TCA-3 exhibited 99.0% similarity to Firmicutes clone B-UP-T0 OTU4 (FM204984) found in a 1,2-DCA-contaminated groundwater. TCA-1 exhibited 99.0% similarity to *Geobacter lovleyi* strain Geo7.1A (JN982204), which was isolated from an anaerobic microbial enrichment culture of organohalide-respiring and could dechlorinate tetrachloroethylene (PCE) and TCA. TCA-2 exhibited 94.0% similarity to uncultured bacterium TfC20L52 (EU362313), which was able to reductively dechlorinate PCE to *trans*-DCE (Kittelmann and Friedrich, 2008). TCA-5 exhibited 99.0% similarity to bacterium BR20 (GQ461628) presented in a trichloroethylene (TCE) anaerobic dechlorinating culture. TCA-4 exhibited 99.0% similarity to clone ccspost2208 (AY133106) that was isolated from a TCE-contaminated site. Analysis of the dominant species, which consisted of anaerobic dechlorinating culture, may indicate the natural attenuation capability of TCA in groundwater.

## Conclusion

To provide a meaningful analysis of the potential effectiveness of natural attenuation, field and laboratory investigations and simulation of transport and transformation processes were conducted for a TCA contaminated site. Investigation of the shallow groundwater identified significant groundwater contamination and preliminary evidence of natural attenuation. The kinetic parameter of adsorption (0.068 m^3^/kg) and the kinetic constant for biotic dehydrochlorination of TCA (*k_TCA_* = 0.0045 d^–1^) determined the effluent BTCs from the column experiments. The effluent BTCs confirmed effective natural attenuation of TCA in the aquifer. The development of the entire contamination plume was predicted; after about 5 years the contaminated area was predicted to diminish and the maximum concentration of TCA to fall below the DIV of 300 μg/L. The stable velocity field of the groundwater, low hydraulic slope, and adsorption factor for contamination transport slowed pollutant diffusion, allowing microbial degradation to occur. This finding could explain the main mechanism of natural attenuation. The bacterial community was identified, *Trichococcus*, *Geobacteraceae*, *Geobacter lovleyi*, *Mucilaginibacter*, and *Arthrobacter*, which showed considerably high TCA-degrading activities. This case study illustrated an effective approach to field, laboratory, and modeling investigations of natural attenuation of CAHs for site risk assessment and remedial engineering applications.

### Conflict of Interest Statement

The authors declare that the research was conducted in the absence of any commercial or financial relationships that could be construed as a potential conflict of interest.
